# Single-Cell RNA Sequencing for Analyzing the Intestinal Tract in Healthy and Diseased Individuals

**DOI:** 10.3389/fcell.2022.915654

**Published:** 2022-07-07

**Authors:** Hua Yan, Yumeng Ye, HanZheng Zhao, Hongyan Zuo, Yang Li

**Affiliations:** ^1^ Department of Experimental Pathology, Beijing Institute of Radiation Medicine, Beijing, China; ^2^ The Seventh Medical Center of PLA General Hospital, Beijing, China; ^3^ Department of General Surgery, The Second Affiliated Hospital of Harbin Medical University, Harbin, China; ^4^ Department of Pathology, Chengde Medical College, Chengde, China; ^5^ Academy of Life Sciences, Anhui Medical University, Hefei, China

**Keywords:** scRNA-seq, intestinal tract, cell subtype, intestinal diseases, enterocytes

## Abstract

The intestinal tract is composed of different cell lineages with distinct functions and gene expression profiles, providing uptake of nutrients and protection against insults to the gut lumen. Changes in or damage to the cellulosity or local environment of the intestinal tract can cause various diseases. Single-cell RNA sequencing (scRNA-seq) is a powerful tool for profiling and analyzing individual cell data, making it possible to resolve rare and intermediate cell states that are hardly observed at the bulk level. In this review, we discuss the application of intestinal tract scRNA-seq in identifying novel cell subtypes and states, targets, and explaining the molecular mechanisms involved in intestinal diseases. Finally, we provide future perspectives on using single-cell techniques to discover molecular and cellular targets and biomarkers as a new approach for developing novel therapeutics for intestinal diseases.

## Introduction

Cells are the smallest structural and functional units of life, and they vary enormously, mainly due to distinct gene expression profiles; thus, almost all organs and tissues are composed of a heterogeneous mixture of cell types. Transcription studies using bulk samples can only measure the average gene expression level in a large population of cells, which is insufficient for studying heterogeneous systems or complex tissues (Wen and Tang, 2016). Since its first use in 2009, single-cell RNA sequencing (scRNA-seq) has been improved considerably during recent years and has been widely applied in stem cell biology, tumor biology, developmental biology, immunology, and targeted drug discovery (Baslan and Hicks, 2017; Ofengeim et al., 2017; Papalexi and Satija, 2017). Single-cell resolution provides insights into cell-specific changes in gene expression, such as cell type or state identification, trajectory inference, and the identification of therapeutic targets and biomarkers.

**FIGURE 1:**
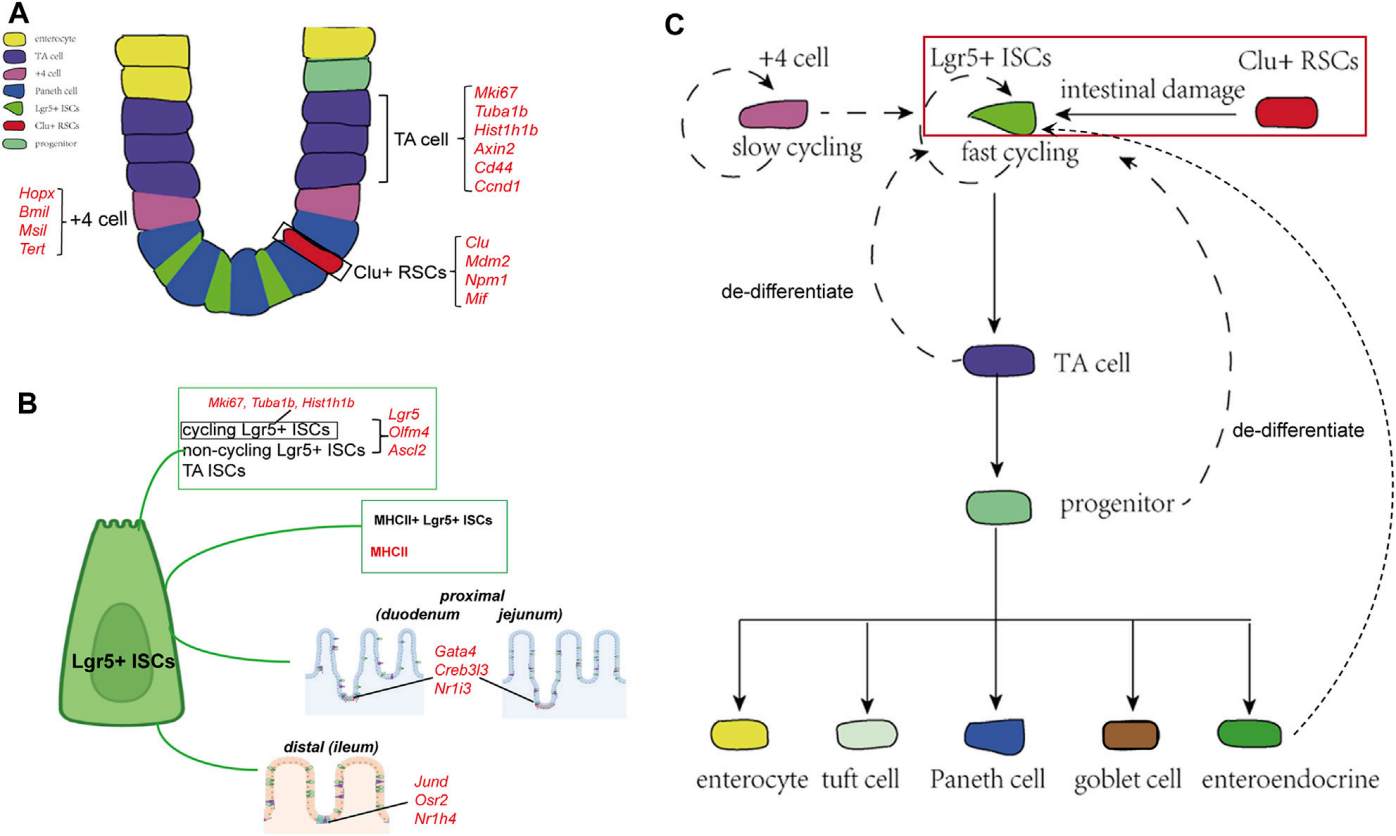
Cellular components and the derivation or differentiation of intestinal epithelium. **(A)** Architecture and cellular components of the intestinal epithelium. The intestinal epithelium folds into millions of crypts and villi. Rapidly dividing ISCs reside at the bottom of the crypt, giving rise to TA cells that finally differentiate into five different mature intestinal epithelial cell types: enterocytes, Paneth cells, goblet cells, EECs, and tuft cells. Clu^+^ RSCs are extremely rare under homoeostatic conditions and can repopulate the pool of Lgr5^+^ ISCs. **(B)** Landmark genes of Lgr5^+^ ISCs based on single-cell sequencing results. Cycling and non-cycling Lgr5^+^ populations strongly expressed canonical CBC genes (Lgr5, Olfm4, and Ascl2). Cell cycle mRNAs (Mki67, Tuba1b, and Hist1h1b) are specifically segregated to cycling Lgr5^+^ cells. Moreover, two Lgr5^+^ ISCs subtypes that specifically expressed MHCII are identified by scRNA-sq. Lgr5^+^ ISCs in different regions of the intestinal tract express region-specific marker genes. **(C)** The derivation and differentiation of ISCs. ISCs in crypts can replenish the whole crypt-villus axis, generating all differentiated cell types required for the physiological function of the intestine.

The intestinal tract comprises different cell lineages, which fold into millions of crypts and villi. Rapidly dividing intestinal stem cells (ISCs) reside at the bottom of the crypt, giving rise to transit-amplifying (TA) cells that finally differentiate into five different mature intestinal epithelial cell types: enterocytes, Paneth cells, goblet cells, enteroendocrine cells (EECs), and tuft cells (Gehart and Clevers, 2018; Spit et al., 2018). In addition, the intestinal tract contains the largest pool of immune cells (such as T cells, macrophages, dendritic cells, and innate lymphoid cells) in the body, which are essential for maintaining mucosal homeostasis in the face of microbial infection and physical and chemical stimulation (Garg et al., 2009; Bain and Mowat, 2014a; Bain and Schridde, 2018; He et al., 2018). Homeostasis imbalance can cause different bowel diseases, such as inflammatory bowel disease (IBD) and colorectal cancer (CRC). However, problems with characterization the phenotypes and origins of different cell populations in the mucosa have impeded defining the biological roles of different cell types in the intestinal tract. Recently, several studies utilizing scRNA-seq focused on the intestinal tract have provided inspiring perspectives in unveiling rare cellular subtypes and differentiation pathways, and revealing previously unexplored cellular functions, which have not been covered in research using bulk samples. Here, we review the current applications of intestinal tract scRNA-seq in identifying novel cell subtypes and states, discovering therapeutic targets, and explaining the molecular mechanisms of intestinal diseases (Figure 1).

## Insights From Single-Cell Analysis of the Intestinal Tract to Discover Novel Subsets

### Intestinal Stem Cells

ISCs that reside at the bottom of crypts can repopulate them to maintain homeostasis and replace the injured epithelium (Spit, et al., 2018; Jasper, 2020). Researchers have identified several ISC populations in the small intestine based on their markers and localization in the crypt, including fast-cycling Lgr5^+^ ISCs, slow dividing +4 cells located at a position far from the crypt base, and reserve stem cells (Barker et al., 2007; Van der Flier and Clevers, 2009; Tian et al., 2011; Yan et al., 2011). Lgr5^+^ ISCs are deemed indispensable for maintaining intestinal epithelial homeostasis and regeneration following injuries, while +4 cells are considered quiescent, slow-cycling stem cells that could repopulate the pool of Lgr5^+^ cells (Montgomery et al., 2011; Tian et al., 2011; Van et al., 2012; Gehart and Clevers, 2018). However, the +4 cell markers are also detected in Lgr5^+^ ISCs, conferring the previous lineage-tracing-based evidence of the +4 cells (Sangiorgi and Capecchi, 2008; Muñoz et al., 2012; Gehart and Clevers, 2018; Spit et al., 2018).

Owing to the development of scRNA-seq, researchers have been able to characterize the features of heterogeneous stem cell populations in a high-throughput and unbiased manner. To explore the heterogeneity of ISCs, Grün and van Oudenaarden sequenced cells from organoids and mouse intestine cells and identified that Lgr5^+^ ISCs represented a homogenous population of cells mixed with a rare population of Paneth and enteroendocrine cells (Grün et al., 2015). In another study, the expression analyses of canonical cap-binding complex genes, proliferation markers, and Wnt signaling pathway targets differentiated the Lgr5^+^ cells into three clusters: cycling Lgr5^+^ ISCs, non-cycling Lgr5^+^ ISCs, and TA ISCs (Yan et al., 2017b). Biton et al. (2018) identified two Lgr5^+^ ISCs subtypes that specifically expressed major histocompatibility complex class II (MHCII), behaving as non-conventional antigen-presenting cells that present antigens to T-helper cells. In turn, cytokines secreted by T-helper cells regulate the proliferation and differentiation of Lgr5^+^ ISCs, in which proinflammatory cytokines (IL-13 and IL-22) promote differentiation and anti-inflammatory cytokines (IL-10) inhibit it. To further explore the regulation behind Lgr5^+^ ISCs replenishment after injuries, researchers sequenced cells from irradiation-damaged intestines and identified a group of revival stem cells, which appeared in irradiated crypts and specifically expressed several genes involved in inflammatory responses, such as *clusterin*. They used genetic lineage-tracing tools to validate the hypothesis and finally confirmed that these revival stem cells were extremely rare under homoeostatic conditions and could repopulate the pool of LGR5^+^ ISCs and replace lost and damaged enterocytes (Ayyaz et al., 2019). In addition to classical ISCs, several populations of progenitor cells, such as secretory progenitors and enterocyte progenitors, with the ability to de-differentiate and revert to stem-like cells have been described; these cells have been observed to replace the lost Lgr5^+^ ISCs upon radiation damage (Buczacki et al., 2013; Tetteh et al., 2016; Yu et al., 2018). With the aid of scRNA-seq, Yan et al. (2017a) defined a group of enteroendocrine lineages representing the signature marker genes *Bmi1* and *Prox1* ^*+*^ that possessed intestinal stem cell activity and were capable of repopulating the damaged intestinal epithelium.

Numerous bioinformatics methods and tools have been designed to analyze scRNA-seq data, enabling the acquisition of more information about lineage divergence in the same cell type (Neu et al., 2017; Kester and Van Oudenaarden, 2018; Potter, 2018). Haber et al. (2017) profiled cells from the duodenum, jejunum, and ileum and revealed a trajectory from Lgr5^+^ ISCs to progenitor and immature enterocytes. They found that Lgr5^+^ ISCs in different regions of the intestinal tract expressed region-specific marker genes, which predicted distinct terminal types of immature enterocytes in the corresponding segments. Similarly, the trajectory paths from the ISCs to the proximal (duodenum and jejunum) and distal (ileum) enterocytes were also distinct.

### Immune Cells

The intestinal immune system involves multiple cell types, which can be further divided into numerous subtypes according to their phenotypes at distinct stages of cell division or different statuses of the local microenvironment (Kamada and Nunez, 2014; Yue et al., 2019; Zhou et al., 2020). Thus, immune cells are ideally suited for single-cell analysis. Previously, flow cytometry, *in situ* hybridization, and fluorescence-activated cell sorting were the most commonly used single-cell analysis tools (Neu et al., 2017; Stubbington et al., 2017); however, these methods can only sort cells roughly by detecting a few definite RNAs and proteins. Capable of qualifying genome-wide expression in thousands of individual cells, scRNA-seq can not only profile immune cells in different conditions and states but can also discover formerly unrecognized functional genes, clarifying the intercellular networks in immune reactions.

### Macrophages

Macrophages are crucial components of the intestinal tract immune system, which modulates inflammation through the secretion of distinct cytokines and the action of phagocytes (Bain and Mowat, 2014b; Bain and Schridde, 2018). However, it is also implicated in chronic pathologies of the intestinal tract such as IBD and colon disease (CD). By sequencing mucosal macrophages in patients undergoing duodenal transplant surgery, Bujko et al. (2018) identified four distinct macrophage subtypes in the human small intestine, representing different replacement cycles and localization in the intestinal mucosa. Compared with their precursors, circulating monocytes and intestinal macrophages showed decreased responsiveness to proinflammatory stimuli but were efficient at the endocytosis of particulate or soluble material, suggesting that circulating monocytes gradually lose their proinflammatory capacity during migration to the intestinal mucosa. Applying scRNA-seq in colonic tissues from patients with CD, Chapuy et al. (2019) identified a group of proinflammatory CD14^+^ macrophages that specifically accumulated in the inflamed colon and could stimulate intestinal inflammatory T-cell responses through the cytokine interleukin-1β (IL-1β) and contribute to CD severity. To clarify the role of commensal microbiota in the maintenance and differentiation of colonic macrophages, Kang et al. (2019) performed scRNA-seq on MHCII^hi^ colon mononuclear phagocytes from both germ-free and specific pathogen-free (SPF) mice and emphasized the unique role of the intestinal microbiota in the development of colon macrophages. They identified seven macrophage subtypes expressing distinct transcription factors or downstream marker genes and discovered two major developmental trajectories from CCR2^+^ monocyte precursors to colon macrophages. They found the total number of colon macrophages in germ-free mice was lower than that in SPF mice. The two major colon macrophage subtypes, CD11c^+^CD206^int^CD121b^+^ and CD11c^−^CD206^hi^CD121b^−^, showed clearly distinct gene expression profiles, differing abilities for carrying out the endocytosis of macromolecules, and different localization within the lamina propria in relation to other macrophage subtypes.

### Innate Lymphoid Cells

Innate Lymphoid Cells (ILCs) are thymus-derived adaptive immune cells that mainly consist of cytotoxic cells and helper-like ILCs (Spits et al., 2013; Vivier et al., 2018). Although they are considered critical regulators of mucosal immunity and tissue homeostasis, the entire landscape of their cellular status and modulation mechanisms remain elusive. By comparing wild-type mice with antibiotic intervention or germ-free mice, Gury-BenAri et al. (2016) characterized the transcriptional identities of intestinal ILCs and the microbiota-mediated regulation of ILC gene expression and evolution. They discovered that ILC population diversity requires constant signaling from the intestinal microbiota. Hummel J applied scRNA-seq to thymic ILC precursors and defined a developmental trajectory that could be tracked based on the sequential expression of *CD122* and *T-bet* genes, revealing the molecular framework for thymic ILC development from NK1.1^−^ ILC precursors (Hummel et al., 2020). Previous studies have suggested that group 2 innate lymphoid cells (ILC2s) play essential regulatory roles in type 2 inflammation. Xu et al. (2019) dissected the cellular diversity and circuit shifts of immune cells under homeostasis and type 2 intestinal inflammation conditions. They found that the expression of the gene encoding α-calcitonin gene-related peptide (*α-CGRP*) was induced in KLRG1^+^ ILC2s during type 2 intestinal inflammation and that α-CGRP antagonized the proliferation of KLRG1^+^ ILC2s, indicating that the expression of α-CGRP maintained KLRG1^+^ ILC2 homeostasis during type 2 intestinal inflammation. The CD4^+^ regulatory T-cell (Treg) subtypes were categorized according to their distinct degree of non-lymphoid tissue phenotype. Treg recruitment to different tissues showed similar pseudotemporal ordering and gene kinetics. However, the priming and the final stages of functional adaptation in non-lymphoid tissues reflected tissue specificity (Miragaia et al., 2019).

### Enterocytes

**FIGURE 2:**
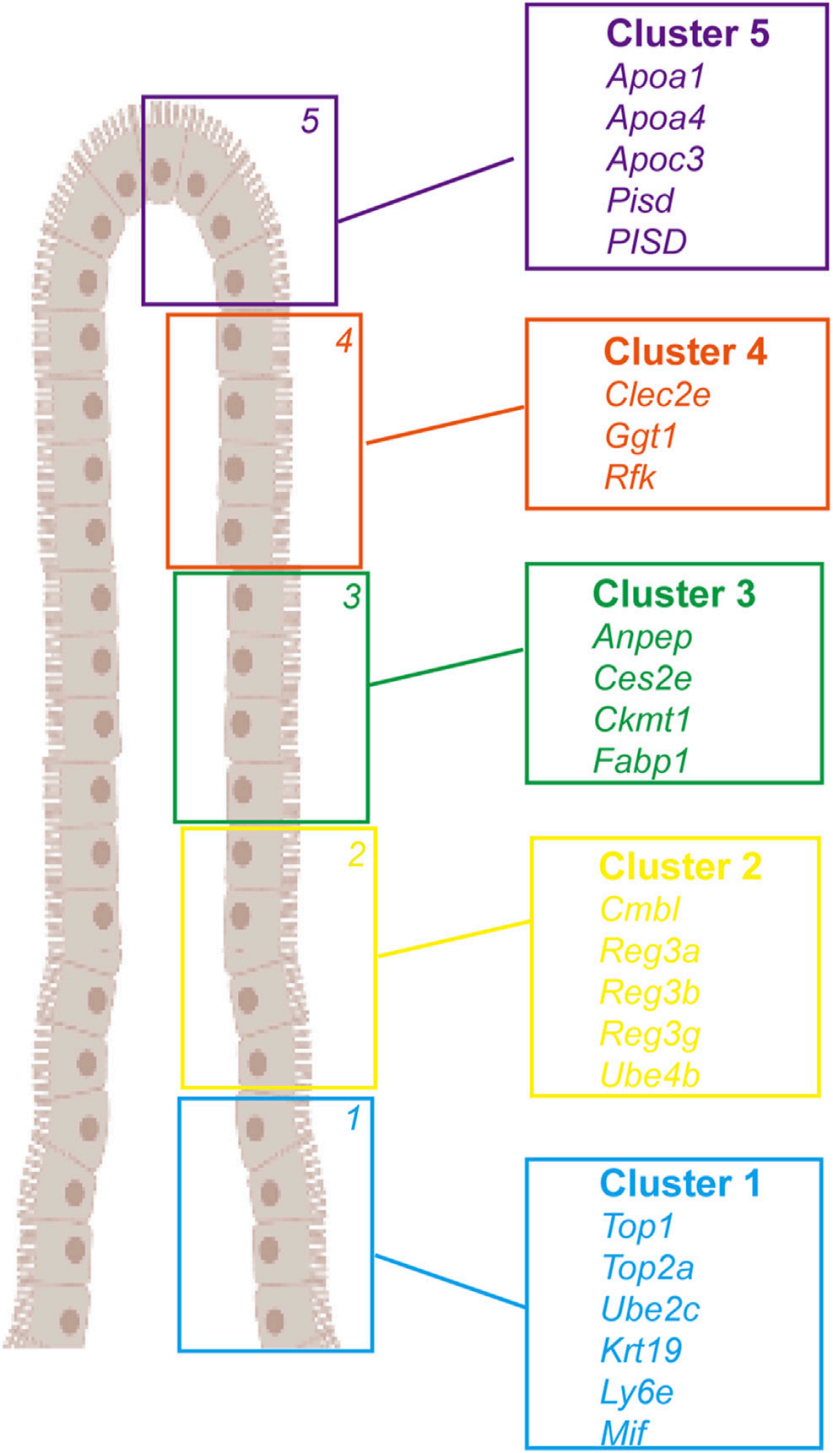
Landmark genes of enterocyte along the villus. A large panel of landmark genes uncovers broad zonation of enterocyte function along the villus. Cluster 1: Top1, Top2a, Ube2c, Krt19, Ly6e, and Mif. Cluster 2: Cmbl, Reg3a, Reg3b, Reg3g, and Ube4b. Cluster 3: Anpep, Ces2e, Ckmt1, and Fabp1. Cluster 4: Clec2e, Ggt1, and Rfk. Cluster 5: Apoa1, Apoa4, Apoc3, Pisd, and PISD).

Enterocytes play an essential role in nutrient digestion and absorption, defense against microbes, and hormone secretion (Van der Flier and Clevers, 2009; Gehart and Clevers, 2018; Spit et al., 2018). Although common cell types of enterocytes have been identified in the mouse intestinal epithelium, their cell type-specific markers and functional characterization data are largely unavailable for the human intestine. Haber et al. (2017) profiled 53,193 individual epithelial cells from the small intestine and organoids of mice and identified several previously unknown enterocyte subtypes based on their gene expression signatures. By sequencing epithelial cells from the human ileum, colon, and rectum, Wang et al. (2020) revealed different expressions of nutrient absorption-related genes in the three segments of the intestinal tract; genes related to the transport of lipids, water, bile salts, and vitamins were highly expressed in the ileum, and individual transporter levels varied in different segments, indicating different nutrient absorption patterns in the small and large intestine. They also identified potential new signature genes for human TA and goblet cells and discovered a new group of cells in the large intestine resembling Paneth cells. Furthermore, the authors compared the human and mouse ileum, observing their cellular landscapes’ common and differential features. Moor et al. (2018) performed a transcriptomic analysis of laser-captured microdissected villus segments to identify landmark genes and localize single-sequenced enterocytes along the villus axis. Based on their position along the villus axis, enterocytes showed sub-specialized and non-overlapping transcription of nutrient transport-related genes and nutrient absorption preferences. The expression of cholesterol transporter genes peaked in the villi tips, peptide transport-related genes were enriched in the upper villus, amino acid and carbohydrate transporters were highly expressed in the middle of the villus zone, and the anti-bacterial *Reg* gene was specifically expressed at the villi bottoms (Figure 2).

In addition to the differences in gene expression and function, scRNA-seq also provides inspired discoveries in the enterocytes’ compositional changes under infectious conditions. Bacterial or helminth infections can affect the composition of epithelial cell types. *Salmonella* infection leads to an increase in the proportion of Paneth cells and absorptive enterocytes, as well as broad activation of the anti-bacterial program, whereas helminth infection causes considerable accumulation of secretory cell types, such as goblet and tuft cells (Haber et al., 2017).

### Enteroendocrine Cells

EECs secrete various hormones, including secretin (Sct), proglucagon (Gcg), neurotensin (Nts), galanin (Gal), glucagon-like peptides, serotonin (Tph1), somatostatin (Sst), cholecystokinin (Cck), ghrelin (Ghrl), gastric inhibitory peptide, and tachykinin, which mainly regulate gastrointestinal activity and metabolic processes (Klok et al., 2007; Furness et al., 2013; Habib et al., 2013; Worthington et al., 2017; Gehart and Clevers 2018). EECs subtypes are generally classified according to the hormones they secrete. Utilizing scRNA-seq, Haber et al. (2017) divided EECs into 12 subtypes by exploring the gene expression profile and regulation of distinct hormone secretion in each subtype. They distinguished between two subtypes of tuft cells (immune and neuronal tuft cells), one of which expresses the epithelial cytokine Tslp and the pan-immune marker CD45, and found that Sct and Cck hormones formerly considered to be produced solely by a particular cell subtype (Sct, Cck, Gcg or GIP are traditionally termed S, I, L, and K cells) were co-produced by multiple EEC clusters. Similarly, combining scRNA-seq with peptide and receptor knock-in line studies. Guo et al. (2019) identified ten major EEC subtypes in the adult *Drosophila* midgut, which produced approximately 14 different hormones in total, and each subtype co-produced two to five different hormones, such as neuropeptide-like precursor 2 (Nplp2) and glycoprotein hormone beta 5 (Gbp5). They determined the spatial distribution of EECs subtypes along the length of the midgut using a region-specific gene enrichment algorithm. By processing subtype-enriched transcription factors (TFs), the authors identified a series of class-specific and region-specific TFs that participate in the EEC subtypes specification, which indicates that EECs diversity was co-regulated by class-specific and region-specific TFs, such as Esg, Drm, Exex, and Fer1. In fact, class-specific TFs were controlled by Notch signaling, and region-specific TFs were specified by anterior-posterior body planning during early cell development. Beumer et al. (2020) established an organoid-based platform for functional studies of human EECs. It partitioned EECs subtypes by noting the key differences in hormone expression, sensory receptors, and TFs profile, concluding that different EECs subtypes carry distinct hormone receptors. In organoids derived from murine Lgr5^+^ ISCs, EEC subtypes showed a more flexible hormone repertoire than was previously proposed. EECs differentially expressed hormone biosynthesis genes between crypts and villi, and bone morphogenetic protein-4 (BMP4) expression altered the hormone profiles of individual EECs by inducing migration of EECs along the crypt-villus axis (Beumer et al., 2018).

In addition to the common cell types, scRNA-seq is also widely applied for identifying rare cells, such as tuft cells and proglucagon-expressing cells. Grun et al. (2015) built a method for rare cell type identification (RaceID) to classify different cell types in a complex mixture. They used RaceID to identify Reg4 as a novel marker for enteroendocrine cells, revealing heterogeneity among rare cells. Haber et al. (2017) distinguished two subtypes of tuft cells. Kalucka et al. (2020) constructed an atlas of EEC transcriptomes from 11 mouse tissues and identified 78 EEC subtypes, including Aqp7^+^ intestinal capillaries and angiogenic EECs. They found that EECs from different tissues express partially overlapping signature genes, which was based on EC markers (Pecam1 and Cdh5), and excluded contaminating smooth muscle cells (Acta2), fibroblasts (Col1a1), and red blood cells (Hba-a1, Hba-a2, and Hbb-bs). Capillary EECs showed more heterogeneity than EECs from arterial, venous, and lymphatic tissues, which exhibited a similar transcriptome profile.

## Single-Cell Sequencing in Intestinal Organoids

Organoids are three-dimensional cell cultures that mimic organ functions and structures. Intestinal organoid models that share approximately 90% of genetic mutations with the natural organ have been developed as versatile *in vitro* platforms for stem cell biology and disease modeling (Almeqdadi et al., 2019; Yoo and Donowitz, 2019). However, it is unclear whether intestinal organoids represent the cellular heterogeneity observed *in vivo*. By carrying out unsupervised hierarchical clustering of scRNA-seq data, Fujii et al. (2018) and Finkbeiner and Spence (2013) showed that human intestinal organoids (HIOs) most closely resembled the human fetal intestine, in which genes related to digestive function and Paneth cell host defense were expressed at low levels. This study also revealed that the ISCs marker OLFM4 expression was limited in the fetal intestine and HIOs, but robust in adult crypts. After *in vivo* transplantation, HIOs became more adult-like, indicating suitability for modeling fetal-to-adult maturation. Chen et al. (2018) used scRNA-seq to investigate the transcriptome of tumor organoids derived from human CRC and revealed that cancer heterogeneity was sustained in tumor organoids. They treated tumor organoids with oxaliplatin and found that the diversity of tumor cell populations in organoids was significantly perturbed, in which the chemosensitive subgroups were depleted and new drug-tolerant subgroups emerged, validating the organoid model’s capability to emulate tumor heterogeneity and response to chemotherapy.

## Single-Cell Sequencing in Bowel Disease to Explore the Underlying Mechanism

### Colorectal Cancer

CRC is the third most common cancer in the world (Yu et al., 2014; Arvelo et al., 2015). Previous pathogenesis studies were difficult to reproduce CRC due to its genome instability, transcriptome alterations, and epigenetic abnormalities (Li et al., 2017; Levitin et al., 2018). Unbiased scRNA-seq provides a unique resolution to characterize aberrant cell states, origin, and development of CRC (Bagnoli et al., 2019; Tieng et al., 2020).

### Intratumor Heterogeneity

Dai et al. (2019) categorized CRC cells into five distinct clusters with different functions using specifically expressed gene signatures. Combining data from single-cell and bulk RNA-seq of CRC samples. Chen et al. (2016) proposed that single-cell analysis not only summarized the single nucleotide polymorphism results of bulk RNA-seq but also screened several fusion transcripts, which were helpful for early diagnosis and determination of the mechanism underlying cancer cell metabolism. They found colon cancer-related pathways with single-cell level SNP enrichment, including the TGF-β and p53 signaling pathways. Within tumor tissue, chemokines play an important role in promoting tumor growth and progression. Adalsteinsson et al. (2013) used quantitative micro-engraving to assess the secretion of ELR^+^ CXC chemokines in CRC, stroma, and adjacent normal tissue, characterized multiple factors including secretion rate and the number of cells secreting each chemokine. They found that the magnitude of ELR^+^ CXC chemokine secretion in cells from CRC and stroma showed polyfunctional heterogeneity, suggesting that the secretory and combination states among CRC cells are complex. Liu et al. (2017) sequenced cells from different regions of the same tumor and observed that genetic or epigenetic alterations were formed early in cancer development, which can be inherited steadily by their offspring, increasing intratumor heterogeneity among different individuals. Zhang et al. (2019a) compared the single-cell transcriptome of epithelial cells from CRC and normal tissues and identified a series of distinct transcripts. Genes related to ribosomes, protein processing in the endoplasmic reticulum, antigen processing and presentation, and the p53 signaling pathway were upregulated in CRC epithelial cells. In contrast, genes related to mineral absorption, aldosterone-regulated sodium reabsorption, and oxidative phosphorylation pathways were specifically downregulated in CRC epithelial cells. Biological analysis of these discriminative transcripts will help reveal the molecular mechanisms implicated in CRC.

### Colorectal Cancer Origins and Clonality

Single-cell sequencing is also a powerful tool for describing clonal relationships and identifying essential genes driving tumor development. Yu et al. (2014) identified two subtypes of CRC cells and tried to explain their origin heterogeneity by detecting mutations prevalent in each subtype. The primary CRC subtype harbored adenomatous polyposis coli (APC) and tumor protein 53 (TP53) mutations, whereas the secondary subtype predominantly contained cell division cycle 27 (CDC27) and poly (A) binding protein cytoplasmic 1 (PABPC1) mutations, suggesting that these two CRC subtypes had different cellular origins. This study also identified a mutated gene, the solute carrier family 12 member 5 (SLC12A5), as a potential cancer driver gene in CRC tumorigenesis, as mutated SLC12A5 could promote cell proliferation in two CRC cell lines. Interestingly, SLC12A5 exhibited a high mutation prevalence at the single-cell level but showed a low mutational frequency at the population level. Liu et al. (2018) compared the somatic copy number alterations (SCNAs) in CRC stem cells and differentiated tumor cells (DTCs) and found that SCNAs in CRC stem cells were more reproducible than in DTCs, indicating that certain mutations in chromosomes are required for CRC development. Notably, similar SCNA patterns in CRC stem cells and DTCs were observed for a single patient, while different patients had specific SCNA profiles.

### Tumor Microenvironment

The tumor microenvironment consists of stromal cells, the extracellular matrix (ECM), structural components, and molecules (Krog and Henry, 2018). Stromal cells include endothelial and immune cells, pericytes, and fibroblasts. ECM and structural components are secreted by both tumor cells and stromal cells (Levitin et al., 2018). The diverse molecules produced by various cell types are critical for the modulation of tumor growth and progression. Immune components have recently attracted wide attention and are considered potential therapeutic targets. Zhang et al. (2020a) profiled the microenvironment composition and characteristics of CRC liver metastases and identified six distinct cell types, including tumor cells, myeloid cells, T cells, B cells, endothelial cells, and fibroblasts. They clustered the regulated genes into six gene groups, namely, FABP4, SPP1, GPNMB, SPARCL1, RBP7, and MGP, and analyzed the pathways in tumors and T cells by conducting functional enrichment analysis.

In CRC, T cells behave as critical elements of the tumor microenvironment. A web-based tool was developed using a comprehensive analysis of the T-cell expression profile and full-length T-cell receptor (TCR) sequencing from peripheral blood, CRC, and adjacent normal tissues. Therefore, this tool enables dissecting the function, development, and migration of CRC T cells using both transcriptome and TCR sequences (Zhang et al., 2019b). Furthermore, scRNA-seq can provide insights into the dynamic relationships among T-cell subtypes within CRC. By examining similarities in the expression of T-cell signature genes, researchers revealed that CD8^+^ effector and exhausted T cells were independently connected with tumor-resident CD8^+^ effector memory cells, and T-regulatory cells were developmentally linked to several T-helper cell subtypes (Zhang et al., 2018). A comprehensive analysis of myeloid cells in human and mouse CRC tissues identified cellular interactions that mediate immunity in CRC (Zhang et al., 2020b). Macrophage and dendritic cell subtypes contribute to cellular crosstalk in the tumor microenvironment, but their responses to myeloid-targeted immunotherapy are distinct. Anti-CSF1R treatment selectively depleted inflammatory macrophages and did not affect macrophages with pro-tumorigenic signatures, whereas CD40 agonist antibody increased Th1-like cells, CD8^+^ memory T cells, and activated the dendritic cell (DC) population. de Vries et al. (2020) discovered a novel innate lymphocyte population in CRC tissues that demonstrated a tissue-resident phenotype and displayed cytotoxic activity. Their infiltration into CRC was correlated with tumor-resident cytotoxic-helper and γδ-T cells with similar phenotypes.

### Inflammatory Bowel Disease

IBD is associated with inappropriate immune responses to commensal microflora in the intestinal tract (Fakhoury et al., 2014; Abegunde et al., 2016). Chapuy et al. (2019) identified six subpopulations within the colon CD14^+^ macrophage population, two of which were monocyte-like CD64^hi^CD163^−^ and macrophage-like CD64^hi^CD163^hi^ cells. CD64^hi^CD163^−^ cells secreted proinflammatory cytokines, such as IL-1β and IL-23, specifically infiltrating the inflamed colon, which correlated with the endoscopic disease severity score. Moreover, CD64^hi^CD163^−^ cells can promote Th17/Th1 responses in tissue memory CD4^+^ T cells through the IL-1β signaling pathway. By comparing the transcriptomes of patients with ulcerative colitis (UC) and healthy individuals, Smillie et al. (2019) identified 51 cellular subtypes, including epithelial, stromal, and immune cells. Among these, BEST4^+^ enterocytes, microfold-like cells, and inflammatory fibroblasts contributed to resistance to anti-TNF treatment. Mapping the risk variants in cell types and pathways identified UC risk genes as cell type-specific and co-regulated within limited cellular subtypes and pathways. Kinchen et al. (2018) observed four colon fibroblast subtypes expressing distinct transcriptional regulators and functional pathways and identified a stem-like SOX6^+^CD142^+^ subpopulation in neighboring crypts. Moreover, a specifically activated TNFSF14^+^ mesenchymal subpopulation was observed in colitis, fueling inflammation by slowing epithelial proliferation and contributing to oxidative stress.

The continuous renewal of the intestinal mucosa is motivated by various stimuli that activate molecular pathways that help provide a proper ISCs niche in the crypt (Gehart and Clevers, 2018; Spit et al., 2018). IBD alters the communication between enterocytes and intestinal immune cells, leading to the development of inflammatory enterocytes, which further drives inflammation (Yamamoto-Furusho, 2007; Frolkis et al., 2013; Silva et al., 2016). IL-22 is believed to be associated with disruption of epithelial replacement in CD (Cella et al., 2008), and scRNA-seq helps us to understand its effect on ISCs and epithelial renewal. In the ileal organoid model, increased IL-22 led to a decreased organoid survival rate but increased proliferation and size in surviving organoids (Zwarycz et al., 2019). The interleukin-22 receptor subunit alpha 1 (IL-22ra1) was expressed only in a subtype of TA and ISCs, and high IL-22 levels resulted in increased proliferation in the TA zone and inhibition of ISCs expansion. The complex role of IL-22 is due to the downregulation of ISC biomarker expression and the self-renewal associated pathway rather than directly impacting the differentiation programs of ISCs. Cytokine blockade has been proposed as a novel therapeutic strategy for CD due to the stimulatory effect of cytokines on inflammation (Fakhoury et al., 2014). However, only a limited number of patients could benefit from this treatment. Researchers have identified a unique cellular module, the GIMATS module, by sequencing inflamed tissues from patients with CD (Martin et al., 2019). The GIMATS module consists of IgG plasma cells, activated T cells, inflammatory mononuclear phagocytes, and stromal cells, driven by distinct network connectivities. The presence of GIMATS in CD was correlated with the failure to achieve durable corticosteroid-free remission during anti-TNF therapy.

### Congenital Intestinal Diseases

Fawkner-Corbett et al. (2021) combined scRNA-seq and spatial transcriptomics to profile the atlas of human intestinal development from 77 intestinal samples that were collected from 17 individual embryos, describing human intestinal morphogenesis across time, location, and cellular compartments, including the principles of crypt-villus axis formation as well as neural, vascular, mesenchymal morphogenesis, and immune populations of the developing gut. By linking these data to rare congenital intestinal disorders, they provided new sights into developmental intestine diseases that are challenging to study *in utero* (spatiotemporal analysis of human intestinal development at single-cell resolution).

## Discussion

The improvement of single-cell technology, increasing gene coverage and stability and reliability, together with the cost reduction of next-generation sequencing technologies, made it possible to dissect the physiological and pathological functions of single-cells (Bacher and Kendziorski, 2016). A new understanding of life forms and biological behavior will occur through a deep understanding of the normal intestine’s cell types, states, action processes, and cooperation mechanisms. In the field of intestinal diseases, scRNA-seq technology has inspired findings from various research perspectives, such as clonal differentiation of cells during disease development, heterogeneity of cells in the intestinal tract, types of cells involved in diseases, changes in immune cells, regulation of gene expression networks, dynamic changes between transcription and protein abundance, and mechanisms of molecular or cell networks in complex tissues.

However, scRNA-seq has several technical limitations for intestinal applications. The gastrointestinal tract has a complex layered structure (Jasper, 2020). The intestinal mucosa, which faces the lumen of the inner intestinal space, is supported by the submucosa and outlined by the longitudinal and circular smooth muscles, which form the muscularis externa (Spit et al., 2018). A single-cell suspension is obtained by mechanical or enzymatic dissociation of the bulk tissue. Furthermore, scRNA-seq requires cells to remain intact after digestion, enrichment, or sorting to prevent RNA degradation prior to library preparation. Thus, obtaining single-cell suspensions containing integral and viable cells without contamination is a challenge. ISCs and epithelial cells are more vulnerable to collagenase treatment, which may lead to an increased number of dead cells and poor quality of the entire sample. Hence, shortening the time spent on single-cell suspension steps significantly improved the overall cell survival in the samples. This should be considered when the experiment involves large groups of animals because the time spent working with multiple samples may double the total length of the experiment. Another drawback of scRNA-seq is data analysis (Potter, 2018). Various computational methods have been developed to analyze intestinal single-cell sequencing data, turn massive data into meaningful breakthroughs to identify distinct cell subtypes, clarify mechanisms, and develop new therapeutical strategies. However, scRNA-seq provides a snapshot of the gene expression profile for each individual cell and lacks standardization for data analysis. Therefore, researchers have attempted to combine scRNA-seq with classical molecular pathology methods and genetic lineage-tracing tools to support the conclusions derived from scRNA-seq. However, an ideal approach to deal with high noise levels, technical variability, and batch effects remains elusive.

Despite the bottleneck associated with single-cell dissociation and data analysis, the application of scRNA-seq in the intestinal tract has resulted in outstanding discoveries. With the development of microfluidic technology in recent years, the number of cells submitted for barcoding and processing for scRNA-seq has increased significantly. As the scale of data collected from transcriptome sequencing continues to grow, together with improvements in analytical methods, scRNA-seq could be a standard tool for discovering pathogenesis, signal networks, and strategies for therapeutics.
